# Should Standardized Time-based Patient Pathways (STP's) be initiated on symptoms or presumptive diagnoses?

**DOI:** 10.1186/1757-7241-20-S2-P45

**Published:** 2012-04-16

**Authors:** Ulrik Frydkjær-Olsen, Rasmus Carter-Storch, Christian Backer Mogensen

**Affiliations:** 1 Emergency Department, Kolding Hospital, Denmark

## Background

STP’s seek to optimize the hospitalization process by letting the patient undergo a package of examinations tailored to the main symptom. This could be of paramount importance for the doctor to diagnose and initiate appropriate treatment and thereby reduce the time of hospitalization. The vital question is whether the chosen STP's ought to be initiated due to the symptoms observed in the patients, or by the presumptive diagnoses made by the admitting General Practitioner (GP).

The aim of the present study was to evaluate the diagnostic value of serious or frequently occurring diagnoses made by the admitting GP.

## Methods

A retrospective cohort study of all registered admissions to Kolding Emergency Department (ED) within a period of one year. The department covers acute medical, orthopaedic, abdominal and vascular surgical admissions to the hospital. The data was retrieved from the electronic interactive white board registration system where the nurses had registered which symptoms or presumptive diagnoses the patients were allocated with by the referring GP.

Ten of the very serious or commonly presented presumptive diagnoses were compared with the action diagnoses made at the time of discharge and the sensitivity, specificity, diagnostic accuracy, positive and negative predictive value were calculated.

## Results

Among the 10,070 patients admitted to Kolding ED during 2010, the presumptive diagnoses acute coronary syndrome, pneumonia, intestinal obstruction, appendicitis, meningitis, deep venous thrombosis, pancreatitis, cholelithiasis, lung embolus and pyelonephritis were compared with the action diagnoses at the hospital. As examples, a total of 1,514 patients had abdominal pain and GP referred 509 of them with appendicitis as the presumptive diagnosis. The diagnostic accuracy was 73% with a sensitivity of 82% and specificity at 72%. 619 patients presented with chest pain of which the GP thought 434 had acute coronary syndrome. The diagnostic accuracy was 34% the sensitivity was 80% and specificity was 31%.

## Conclusion

The diagnostic value of ten presumptive diagnoses revealed that in some situations the assessment from the GP added significant information to the STP's, which directed the attention of the ED physician to certain conditions, whereas in other cases, the information was less usable.

